# Xuebijing exerts protective effects on lung permeability leakage and lung injury by upregulating Toll-interacting protein expression in rats with sepsis

**DOI:** 10.3892/ijmm.2014.1943

**Published:** 2014-09-23

**Authors:** MING-WEI LIU, YUN-HUI WANG, CHUAN-YUN QIAN, HUI LI

**Affiliations:** 1 Department of Emergency, The First Hospital Affiliated To Kunming Medical University, Kunming, Yunnan 650000, P.R. China; 2 Surgical Intensive Care Unit, The Second Hospital Affiliated To Kunming Medical University, Kunming, Yunnan 650000, P.R. China

**Keywords:** sepsis, lung permeability, Xuebijing, Toll-interacting protein, interleukin-1 receptor-associated kinase 1, oxidative stress, rats

## Abstract

Xuebijing (XBJ) is a type of traditional Tibetan medicine, and previous pharmacological studies have shown that the ethanol extract is derived from Chuanxiong, Chishao, Danshen and Honghua. Chuanxiong, Chishao, Danshen and Honghua possesses potent anti-inflammatory activity, and has been used in the treatment of inflammatory infectious diseases. In the present study, we investigated the effects of XBJ on pulmonary permeability and lung injury in cecal ligation and puncture (CLP)-induced sepsis in rats. A CLP sepsis model was established for the control and treatment groups, respectively. Approximately 2 h prior to surgery, an amount of 100 mg/kg XBJ injection was administered to the treatment group. Reverse transcription polymerase chain reaction (PT-PCR) and western blot analysis were used to examine the expression of Toll-interacting protein (Tollip), interleukin-1 receptor-associated kinase 1 (IRAK1), Toll-like receptor 4 (TLR4), nuclear factor-κB65 (NF-κB65) and TNF receptor-associated factor 6 (TRAF6) in lung tissue. ELISA was applied to detect changes of tumor necrosis factor-α (TNF-α), interleukin-6 (IL-6), interleukin-1 (IL-1), interleukin-4 (IL-4) and interleukin-10 (IL-10) levels in bronchoalveolar lavage (BAL) fluid, and intercellular adhesion molecule 1 (ICAM-1) and von Willebrand factor (vWF) in serum. The number of neutrophils, albumin and total cells in the BAL fluid were measured. For histological analysis, hematoxylin and eosin (H&E) stains were evaluated. Lung permeability, the wet/dry weight ratio (W/D) and the lung pathology score were determined following the induction of ALI by CLP for 24 h. The results demonstrated that XBJ upregulated Tollip expression and blocked the activity of IRAK1, TLR4, NF-κβ65 and TRAF6. Additionally, the number of neutrophils and total cells were significantly decreased in the XBJ group compared to that in the control group. Lung permeability, the wet/dry weight ratio (W/D) and the lung pathology score were significantly decreased in the XBJ group. The histological results also demonstrated the attenuation effect of XBJ on CLP-induced lung inflammation. The results of the present study indicated that XBJ has a significantly reduced CLP-induced lung permeability by upregulating Tollip expression. The protective effects of XBJ suggest its therapeutic potential in CLP-induced acute lung injury treatment.

## Introduction

Sepsis, a systemic inflammatory response syndrome (SIRS) caused by infection, is confirmed to be accompanied with the presence of bacteria or highly suspicious focus of infection. Sepsis can easily cause acute lung injury (ALI) (1,2). When ALI occurs, cytokines, chemokines, adhesion molecules and other inflammatory mediators are produced in the endothelial cells within activated pulmonary vascular cells, which destroy the integrity of pulmonary vascular endothelial cells and lead to the increase of blood capillary permeability of the lung. This, in turn, causes pulmonary edema (3), resulting in acute respiratory distress syndrome, multiple organ failure and high mortality, presenting issues for carers in the Intensive Care Unit (ICU).

Toll-interacting protein (Tollip) is an interactor of the Toll family of proteins, which are highly conserved in evolution from *Drosophila* to humans, and include the Toll-like and interleukin-1 (IL-1) receptors, which are involved in the inflammatory response. Tollip is involved in two main functions. The first, suggested by Burns and collaborators (4), identifies Tollip as an interactor of the IL-1 receptor TIR domain, mediates the binding of the serine/threonine kinase IRAK-1 to the activated receptor complex, making it an integral component of the IL-1RI signaling cascade. In their study, Yamakami and Yokosawa (5) identified a negative regulatory role of Tollip on the IL-1β and TNF-α signaling pathways, which is in agreement with the inhibition of NF-κB activation observed following Tollip overexpression (4). The second function, described by Yamakami *et al* (6), concerns the interaction of Tollip with Tom1, ubiquitin and clathrin in a high molecular mass complex involved in protein sorting. In agreement with findings of that study, an endosomal function of the protein was suggested by Katoh *et al* (6,7). Brissoni *et al* (8) showed that Tollip is required in the sorting of the IL-1RI at late endosomes, further clarifying the involvement of Tollip in the IL-1 inflammatory pathway. Zhang and Ghosh (9) demonstrated that Tollip is associated with IL-1RI and the TLR2 and TLR4 receptors when activated by LPS stimulation. This interaction results in the suppression of TLR-mediated cell responses through inhibition of the phosphorylation and kinase activity of IRAK1. Active IRAK1 subsequently causes the dimerization and polyubiquitination of TRAF6, ultimately leading to the production and release of multiple cytokines via NF-κB activation (10). However, *in vivo* murine knockout models have demonstrated that Tollip induced proinflammatory pathways, in contrast to *in vitro* experiments (11).

Xuebijing is a Chinese herb compound preparation mainly comprising Chuanxiong (*Rhizoma Chuanxiong*), Chishao (*Radix Paeoniae Rubra*), Danshen (*Radix Salviae Miltiorrhiae*) and Honghua (*Flos Carthami*). Xuebijing can clear toxic heat, cool blood, promote qi and blood circulation, remove toxic substances and relieve pain (12). Furthermore, Xuebijing has been used to treat systemic inflammatory response syndrome, pyemia and multiple organ dysfunction syndrome. However, the underlying mechanism of action of Xuebijing remains to be determined.

In the present study, we hypothesized that the function of Xuebijing would reduce inflammatory-induced pulmonary vascular permeability by upregulating Tollip expression. We examined the expression of cytokines and Tollip in the lung following treatment with Xuebijing in a rat model of CLP-induced lung injury.

## Materials and methods

### Drug

The Xuebijing injection was purchased from Tianjin Chase Sun Pharmaceutical Co., Ltd. (Tianjin, China; no. Z20040033) and comprised Chuanxiong, Chishao, Danshen and Honghua. Chuanxiong, Chishao, Danshen and Honghua were provided by Professor Li Shixia of the Central South University and deposited in the pharmacy centre.

### Mice

Male Sprague-Dawley mice were purchased from the Kunming Medical University Laboratory Animal Center (Kunming, China). The mice were housed in the Kunming Medical University Animal Care Facility and were maintained in pathogen-free conditions. The mice were 8–9 weeks of age at the initiation of the experiment and were maintained on standard laboratory chow and water *ad libitum*. The experimental protocols were approved by the Committee of Animal Experimentation of the Kunming Medical University, and by the Animal Experimentation Ethics Committee, Kunming Medical University (approval no. 09/060/MIS).

### Reagents

A reverse transcription reaction kit was purchased from Takara Biotechnology Co., Ltd. (Dalian, China). TRIzol and electrophoresis reagents were from ProMag Co., Ltd. (Ningbo, China). The RT reaction kit was obtained from Takara Biotechnology Co., Ltd. The PCR amplification reagent kit and DNA ladder/marker were obtained from Shanghai Sangon Biological Engineering Co., Ltd. (Shanghai, China). GAPDH was obtained from Santa Cruz Biotechnology, Inc. (Santa Cruz, CA, USA). Rabbit anti-mouse Tollip, TLR4, TRAF6, p-IRAK1, VEGF-α, HO-1 and NF-κB polyclonal antibodies were purchased from Wuhan Boster Biological Technology, Ltd. (Wuhan, China). Rabbit anti-mouse IRAK1 and phosphorylated IRAK1 were purchased from Cell Signaling, Technology (Beverly, MA, USA). FITC-albumin, hexadecyl-trimethyl-ammonium bromide were purchased from Sigma-Aldrich (St. Louis, MO, USA). SYBR-Green I was obtained from Biotium (Hayward, CA, USA). The Oligo(dT)18 and primers were synthesized by Shanghai Invitrogen Biotechnology Co., Ltd (Shanghai, China). The dNTP was obtained from Promega (Madison, WI, USA).

### Animal model of sepsis

Studies were performed on rats with an average weight of 40.4 g. To induce sepsis, the rats were anesthetized with isoflurane (4% induction and 2% maintenance) and placed on a warming pad. Following laparotomy, the cecum was exteriorized, and the membrane between the cecum and the mesentery was carefully cut to release the cecum. The cecum was ligated 1.5 cm from the tip or just below the ileocecal valve with 4-0 silk. Two punctures were made with an 18-gauge needle, and 1 mm of fecal material was expressed from the punctures. The incision was sutured in two layers with 4-0 silk. In sham pups, the cecum was located but not ligated or punctured. The animals were resuscitated with 3 ml/100 g body wt normal saline subcutaneously immediately after surgery.

### Grouping and treatment

According to a random number table, 88 rats were randomly divided into 4 groups (n=22 rats per group): normal control group, sham operation group (sham group), sepsis model group (model group) and melilotus treatment group (treatment group). The model and treatment groups were induced by cecal ligation and puncture (CLP) administered via tube into the melilotus extract at a volume of 25 mg/kg every 8 h. The normal control, sham and control groups were administered the same volume of normal saline. Twenty-two rats in each group were anesthetized using ether at specific time-points 24 h post-injection. Blood was collected via the orbital sinus. Ethylenediaminetetraaceticacid (EDTA) was used as an anti-coagulant, and the plasma was isolated by centrifugation at 10,000 × g for 5 min. Lung tissues were washed with saline solution, dried with filter paper and weighed. The plasma and tissues were stored at −20°C for subsequent experiments.

### RNA extraction

For the isolation of RNA tissue, rats were humanely sacrificed under ether anesthesia and aseptic conditions. The lung tissues were removed and immediately frozen in liquid nitrogen. Prior to RNA extraction, the lung samples were homogenized in TRIzol™ reagent (Invitrogen) using Mixer 301. Total RNA was extracted according to the manufacturer’s instructions. RNA samples were electrophoresed in agarose gels and visualized with ethidium bromide for quality control.

### cDNA synthesis and real-time quantitative PCR

RNA (3 μg) was reverse-transcribed with reverse transcriptase for 1 h at 37°C for synthesis of cDNA. Quantitative changes in mRNA expression were assessed with real-time quantitative PCR (Bio-Rad CFX) using SYBR-Green detection consisting of SYBR-Green PCR Master Mix (Aria-tous, Iran). The PCR Master Mix was comprised 0.5 units of Taq polymerase, 2 μl of each primer and 3 μl of each cDNA samples in a final volume of 20 μl. Amplifications were repeated three times. Oligonucleotide primer sequences are provided in Table I. β-actin was used as an endogenous control and each sample was normalized on the basis of its β-actin content. The relative quantification of the mRNA expression levels of target genes was calculated using the 2^−ΔΔCt^ method [Celik et al (13)] (Table II). ΔΔCt = (Ct gene studied − Ct β-actin) treated − (Ct gene studied − Ct β-actin) control.

### Western blot analysis

Lung tissues were snap frozen in liquid nitrogen, pulverized and resuspended in ice-cold lysis buffer (Solarbio, Beijing, China). Protein concentrations were determined with the Bradford method. Lysates were allowed to solubilize on ice for 30 min, and particulate mass was removed by centrifugation at 15,000 × g for 15 min at 4°C. Supernatants were analyzed by SDS-PAGE. Primary antibodies used included rabbit anti-Tollip monoclonal antibody (1:400), rabbit anti-NF-κB monoclonal antibody (1:400), rabbit anti-IRAK1 monoclonal antibody (1:400), rabbit anti-TLR4 monoclonal antibody (1:400), rabbit anti-TRAF6 monoclonal antibody (1:400), rabbit anti-VEGF-α monoclonal antibody (1:400), rabbit anti-Nrf2 monoclonal antibody (1:400), mouse anti-GAPDH monoclonal antibody (1:400) were purchased from Santa Cruz Biotechnology, Inc.. Secondary antibodies were horseradish peroxidase-labeled antibodies (Thermo Scientific Pierce, Rockford, IL, USA). Blots were processed for enhanced chemifluorescence using a Pierce ECL Western blotting substrate (Thermo Scientific Pierce).

### Immunohistochemistry

Immunostaining was performed on lung sections after antigen retrieval using Retrievagen A (Zymed Laboratories Inc., South San Francisco, CA, USA) at 100°C for 20 min, and quenching endogenous peroxidases with 3% H_2_O_2_. Sections were blocked with 2% BSA in PBS followed by staining with primary anti-Tollip (BD Pharmingen, San Jose, CA, USA) at RT for 1 h. The sections were washed, and after application of the secondary antibody (R&D Systems) tissues were developed using Vectastain ABC (Vector Laboratories Inc., Burlingame, CA, USA) and 3,3′-diaminobenzidine (Vector Laboratories). After staining, five high-power fields (x200) were randomly selected on each slide, and the average proportion of positive expression in each field was counted using the true color multi-functional cell image analysis management system (Image-Pro Plus; Media Cybernetics, Rockville, MD, USA) and expressed as a positive unit (pu).

### Cytokine and VEGF measurements in bronchoalveolar lavage and plasma

Mice were sacrificed after 24 h of treatment, and bronchoalveolar lavage (BAL) was performed via the tracheal catheter in the right lung lobes using 0.8 ml of phosphate-buffered saline. The withdrawn fluid was centrifuged at 15,000 × g, and the supernatant was snap frozen and stored at −80°C for further use. Aliquots of BAL fluid and plasma were detected in duplicate with an enzyme-linked immunosorbent assay (ELISA kit offered by Glory Science Co., Ltd. (Del Rio, TX, USA) kits for tumor necrosis factor-α (TNF-α), interleukin (IL)-1β, IL-6 and IL-10 according to the manufacturer’s instructions.

### Measurement of O_2_^−^ in homogenates

Lung tissue (~200 mg of tissue) was placed in 1 ml of ice-cold (4°C) Krebs HEPES buffer, cut with a pair of scissors and homogenized using a pre-cooled Ultra-Turrax homogenizer (3×10 sec bursts). The crude homogenate was incubated for 30 min at 37°C in Krebs HEPES buffer containing 1,000 mg F′ collagenase, 125 mg F′ elastase, 1,000 mg F′ aprotinin and 250 mg F′ trypsin inhibitor. Crude homogenate was washed twice with Krebs HEPES buffer to remove collagenase (centrifuged at 500 × g for 10 min). The pellet was resuspended in 1 ml Krebs-HEPES buffer with a motor driven glass-Teflon homogenizer (3×10 sec), and centrifuged at 12,000 ×g for 20 min in order to remove mitochondria, after which the supernatant was centrifuged at 100,000 × g for 60 min. The final pellet was then resuspended in phosphate-EGTA buffer [mM: 50 phosphate buffer (pH 7.0); 1 EGTA and 100 sucrose]. In the chemiluminescence experiments, 50 μl of resuspended pellet or supernatant was added to 390 μl phosphate-EGTA buffer with 10 μl lucigenin (0–23 mM) in the presence or absence of 50 μM NADH or NADPH (100 μM). The protein content was measured according to the method of Lowry *et al* (14).

### MPO activity determination

MPO activities were determined using an MPO kit produced by Jiancheng Bioengineering Institute (Nanjing, China) according to the manufacturer’s instructions. Briefly, frozen lung samples, were thawed and homongenized in ice-cold buffer provided in the kit. The homogenates were centrifuged at 5,000 × g for 10 min. Pellets were suspended in 0.5% hexadecyl trimethyl ammonium bromide in 50 mM PBS (pH 6.0) and incubated at 60°C for 2 h. After another centrifugation (1,200 × g), supernatants were collected. Their protein concentrations were measured using a protein assay kit (A045; Jiancheng Bioengineering Institute). In a 96-well plate, 15 μg protein was incubated with 100 μl 3,3R,5,5R-tetramethylbenzidine for 3 min. After 100 μl sulphuric acid (1 N) was added, absorbance was read in a spectrophotometer (Metash Instruments Co., Ltd., Shanghai, China) using a wavelength of 450 nm. Original MPO value was normalized with protein contents.

### Superoxide dismutase assay (SOD)

SOD activity was estimated by the method of Kakar *et al* (15). The reaction mixture of this method contained: 0.1 ml of phenazine methosulphate (186 μmol), 1.2 ml of sodium pyrophosphate buffer (0.052 mmol; pH 7.0) and 0.3 ml of the supernatant after centrifugation (1,500 × g for 10 min followed by 10,000 × g for 15 min) of the homogenate was added to the reaction mixture. The enzyme reaction was initiated by adding 0.2 ml of NADH (780 μmol) and stopped after 1 min by adding 1 ml of glacial acetic acid. The amount of chromogen formed was measured by recording color intensity at 560 nm. Results are expressed in U/mg protein.

### Measurement of malondialdehyde (MDA)

MDA was quantified as thiobarbituric acid reactive substances (TBARS) according to previously published methods (16,17) as a measure of lipid peroxidation. Briefly, the weighed samples were homogenized in 1 ml 5% trichloroacetic acid. Samples were centrifuged and 250 ml of the supernatant was reacted with the same volume of 20 mM thiobarbituric acid for 35 min at 95°C, followed by 10 min at 4°C. Sample fluorescence was read using a spectrophotometric plate reader with an excitation wavelength of 515 nm and an emission wavelength of 553 nm.

### Determination of inflammatory cell count in bronchoalveolar lavage (BAL)

BAL was performed by instilling 0.9% NaCl containing 0.6 mmol/l ethylenediaminetetraacetic acids in two separate 0.5 ml aliquots, as previously described (18,19). The fluid was recovered by gentle suction and placed on ice for immediate processing. An aliquot of the BAL fluid was processed immediately for total and differential cell counts. The remainder of the lavage fluid was centrifuged and the supernatant was removed aseptically and stored in individual aliquots at −70°C. Total cell counts in BAL fluid were determined using a haemocytometer. A number of different inflammatory cells was calculated as the percentage of various inflammatory cells multiplied by the total number of cells in the BAL fluid sample. All the analyses were performed in a blinded manner.

### Albumin concentration of the BAL

The albumin content of the BAL supernatants was assessed using an ELISA kit for Albumin (E91028Mu; Uscn Life Science, Wuhan, China). Measurement of the absorbance at 450/540 nm was performed with a microplate reader (Infinite 200; Tecan Group, Männedorf, Switzerland).

### Pulmonary vascular permeability assays

Two hours prior to euthanasia, FITC-labeled albumin (5 mg/kg body wt) was administered via a tail-vein injection at 6 and 24 h. Immediately after euthanasia, the lungs were lavaged three times with phosphate-buffered saline (0.5 ml per lavage) and the samples were combined. Fluid recovery was roughly 95%. The BAL samples were centrifuged at 3,000 × g for 10 min. FITC fluorescence in the BAL fluid was measured using a fluorescence spectrophotometer with excitation at 484 nm and emission at 510 nm.

### Wet/dry lung weight ratio and the water content

A wet-to-dry weight ratio was used as an index of tissue water content. After 24 h of melilotus extract, the animals were anesthetized using ketamine (80 mg/kgip) and xylazine (20 mg/kgip), sacrificed and lungs were excised en bloc. The different lung lobes were cut, blot dried and placed on preweighed glass plates. The wet weight of the tissue was registered immediately. The tray with the tissue was then baked in a hot air oven at 55°C for 72 h to obtain a constant weight. After the dry weight of the tissue was registered, the wet/dry lung weight ratio was calculated as wet weight and dry weight ratio of lung tissue. Lung water content was calculated as the wet weight minus dry weight and wet weight ratio of lung tissue multiplied by 100%.

### Arterial blood gas analysis

Abdominal arterial blood samples (1.5 ml) were obtained at the indicated time-points after 0.9% NaCl, LPS and 250 ppm CO challenge to analyze blood gas. COHb, serum lactate, partial pressure of arterial oxygen (PaO2), and saturation of arterial oxygen (SaO2) were measured using a blood gas analyzer (Roche OMNI S6, USA).

### Pathological observation of lung tissues

The middle lobe of the right lung was fixed by infusing 10% formaldehyde solution in the same pressure, and the inflation of lung was kept uniform and then, embedded in paraffin wax, cut into sections and stained with hematoxylin and eosin (H&E). Pathological tissue changes in tissues were observed using optical microscopy. Lung injury, based on the infiltration of inflammatory cells, pulmonary interstitial and alveolar edema, damage to alveolar structure and degree of fibrosis was assessed using the grading system reported by Szapiel *et al* (20). And ALI was scored as follows (18): i) alveolar congestion, ii) hemorrhage, iii) infiltration or aggregation of neutrophils in airspace or vessel wall, and iv) thickness of alveolar wall/hyaline membrane formation. Each item was scored on a 5-point scale as follows: 0, minimal damage, 1, mild damage, 2, moderate damage, 3, severe damage, and 4, maximal damage. Repeated-measures data were statistically analyzed using repeated-measures analysis of variance (ANOVA).

### Statistical analysis

Statistical analysis was performed with the Statistical Package for the Social Sciences version 15.0 (IBM Corp., Armonk, NY, USA). Data were analyzed for normality using the Kolmogorov-Smirmov method, and the normally distributed data were expressed as mean ± standard deviation. To compare normally distributed data between each group, one-way analysis of variance followed by the Student-Newman-Keuls post hoc test was employed. P<0.05 was considered to indicate a significant result.

## Results

### XBJ administration upregulates the expression of Tollip gene and protein, and blocks pro-inflammatory gene and protein in lung tissue

**Figure 1:**
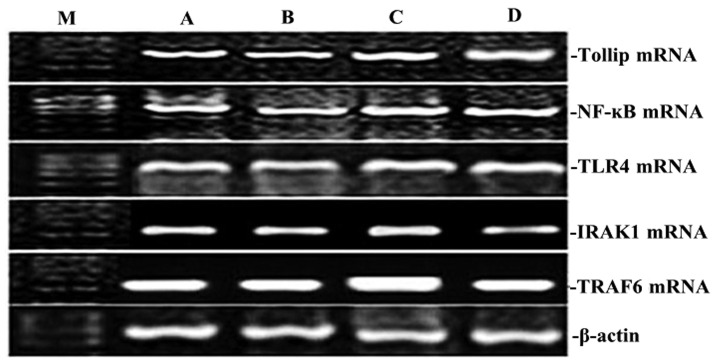
Effect of XBJ on the mRNA expression of Tollip, IRAK1, TLR4, NF-κB65 and TRAF6 in lung tissue. Groups of mice were challenged with CLP and treated with XBJ 24 h later. The expression of Tollip, IRAK1, TLR4, NF-κB65 and TRAF6 in lung tissue was determined by RT-PCR. Representative RT-PCR shows the level of Tollip, IRAK1, TLR4, NF-κB65, and TRAF6 expression in the four rat groups. M, marker; A, normal control group; B, sham operation group; C, control group; D, treatment group.

**Figure 2:**
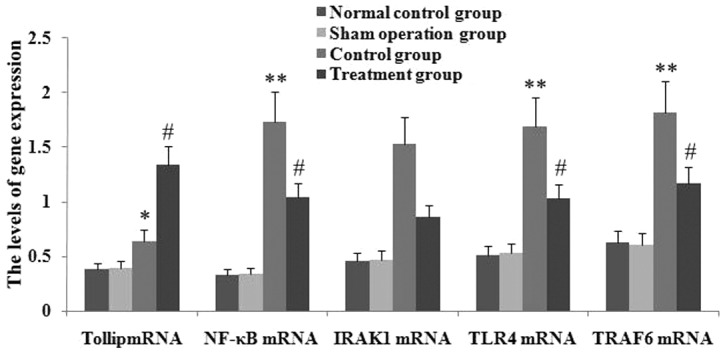
Administration of XBJ led to increased expression levels of Tollip mRNA, and inhibition of TLR4, NF-κB65 and TRAF6 mRNA expression in lung tissue in CLP-ALI mice. The expression of Tollip, IRAK1, TLR4, NF-κB65 and TRAF6 in lung tissue was determined by RT-PCR. Statistical summary of the densitometric analysis of Tollip, IRAK1, TLR4, NF-κB65 and TRAF6 expression in the four rat groups. Data are presented as mean ± standard deviation of one experiment consisting of three replicates. Experiments were performed in triplicate; ^*^P<0.05 and ^**^P<0.01 vs. normal control group and sham operation group. ^#^P<0.05 and ^##^P<0.01 vs. control group.

**Figure 3:**
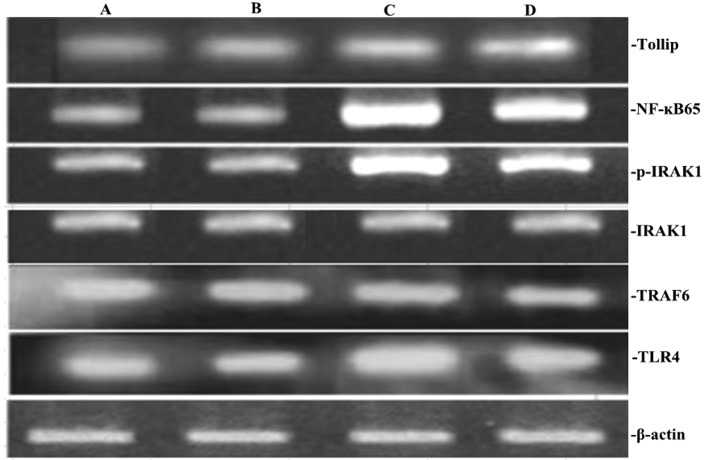
Administration of XBJ enhanced the expression of Tollip protein, and inhibition of TLR4, NF-κB65, p-IRAK1 and TRAF6 protein expression in lung tissue in CLP-ALI mice. Groups of mice were challenged with LPS and treated with salidroside 24 h later. Tollip, p-IRAK1, TLR4, NF-κB65 and TRAF6 were assayed by western blot analysis. Representative western blots show the level of Tollip, p-IRAK1, TLR4, NF-κB65 and TRAF6 protein expression in the four rat groups. A, normal control group; B, sham operation group; C, control group; D, treatment group.

**Figure 4:**
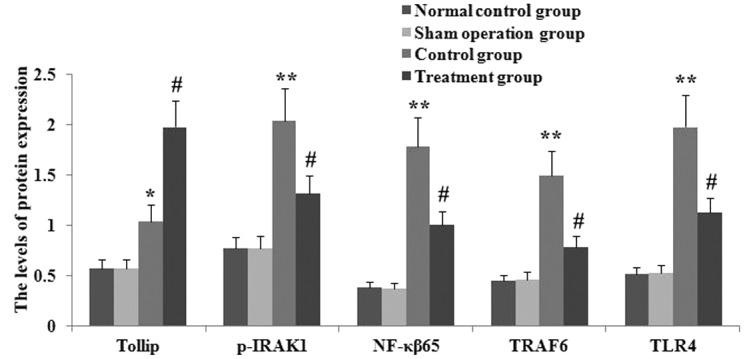
Administration of XBJ enhanced the expression of Tollip protein protein, and inhibition TLR4, NF-κB65, p-IRAK1 and TRAF6 protein expression in lung tissue in CLP-ALI mice. Groups of mice were challenged with LPS and treated with salidroside 24 h later. Tollip, p-IRAK1, TLR4, NF-κB65 and TRAF6 were assayed by western blot analysis. Statistical summary of the densitometric analysis of Tollip, p-IRAK1, TLR4, NF-κB65 and TRAF6 protein expression in the four rat groups. Data are presented as mean ± standard deviation of one experiment consisting of three replicates. Experiments were performed in triplicate; ^**^P<0.01 vs. the normal control group and sham operation group. ^#^P<0.05, ^##^P<0.01 vs. the control group.

To clarify the effect of XBJ on Tollip, TLR4, TRAF6, p-IRAK1 and NF-κB expression induced by CLP, the expression of Tollip, TLR4, TRAF6, p-IRAK1 and NF-κB was measured by RT-PCR and western blotting, respectively. The mRNA and protein expression levels of Tollip, TLR4, TRAF6, p-IRAK1 and NF-κB in rat lung showed significant increases by CLP-induced ALI (P<0.05; Figs. 1–4). However, compared with those by CLP-induced ALI, the mRNA and protein expression levels of Tollip were significantly increased with the administration of XBJ, and TLR4, TRAF6, p-IRAK1 and NF-κB gene and protein expression was significantly decreased (P<0.05; Figs. 1–4).

### XBJ administration increases lung localization of positive Tollip protein in CLP-induced acute lung injury

**Figure 5:**
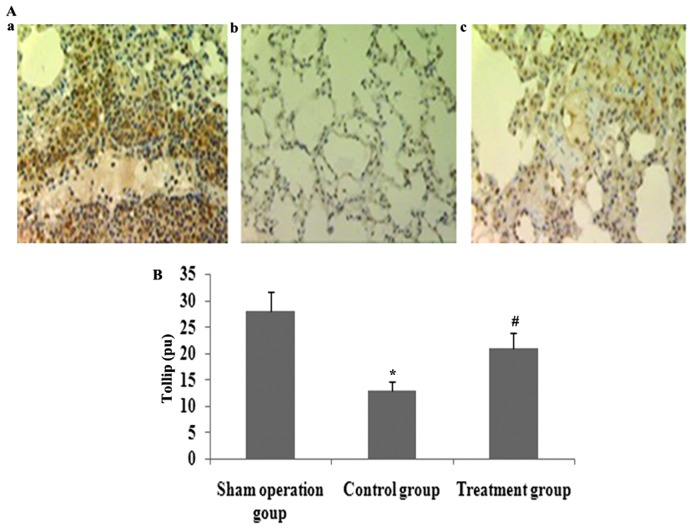
Administration of XBJ upregulated Tollip positive protein in lung tissue in CLP-ALI mice. Groups of mice were challenged with CLP and treated with XBJ 24 h later. Tollip positive protein levels of lung tissue were determined using immunohistochemistry and the average proportion of positive expression in each field was counted using the true color multi-functional cell image analysis management system. Values are expressed as mean ± SD; ^**^P<0.01 vs. normal control group and sham operation group. ^#^P<0.05 and ^##^P<0.01 vs. control group.

Immunohistochemical analysis was used to determined the lung distribution of positive Tollip protein in rat lung 24 h after CLP or saline treatment. Positively immunostained cells appeared brown. The expression of Tollip was specifically localized to the alveolar epithelium. The number of cells expressing Tollip was significantly decreased in CLP-induced acute lung injury, and was markedly increased by XBJ treatment (Fig. 5).

### XBJ administration inhibits MPO activity and inflammatory cell infiltration in lung tissues

**Figure 6:**
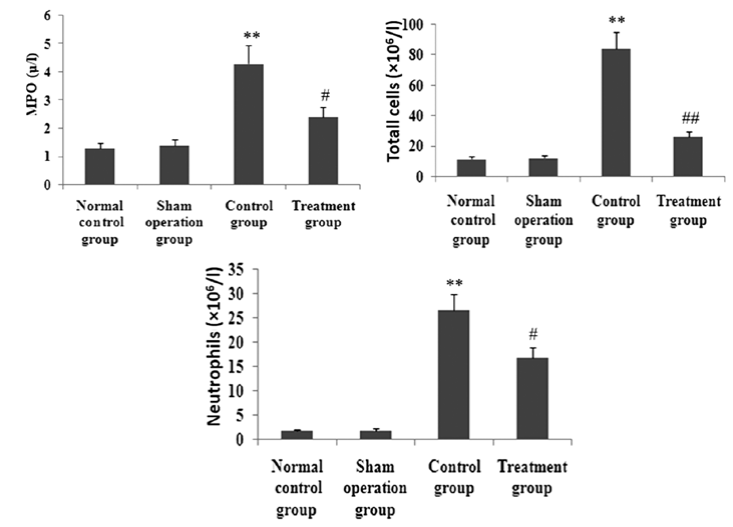
Administration of XBJ attenuated LPS-induced pulmonary inflammatory cell infiltration in lung tissue in CLP-ALI mice. Groups of mice were challenged with CLP and treated with XBJ 24 h later. MPO activity, total cell and neutrophil count were measured. Values are expressed as mean ± SD, ^*^P<0.05, ^**^P<0.01 vs. normal control and sham operation groups; ^#^P<0.05 vs. control group.

To observe the effect of XBJ on MPO activity and inflammatory cell infiltration in lung tissues, MPO activity in lung tissue and inflammatory cells in BAL fluid was determined. As shown in Fig. 6, after animals received CLP, MPO activity in lung tissue and total cells and neutrophils in BAL fluid was markedly enhanced. However, the increase of MPO activity in lung tissue and total cells and neutrophils in BAL fluid was markedly inhibited by XBJ.

### XBJ administration decreases CLP-induced lung inflammatory response

**Figure 7:**
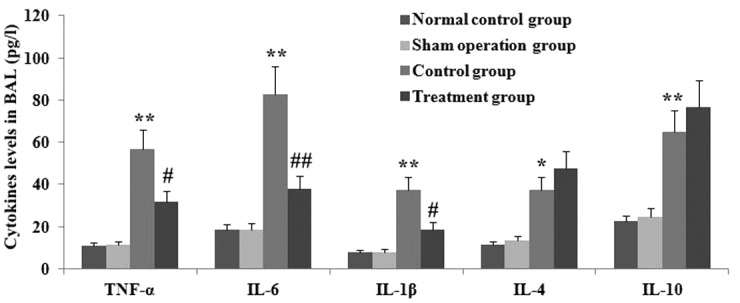
Administration of XBJ attenuated LPS-induced pulmonary inflammation. Groups of mice were challenged with CLP and treated with XBJ 24 h later. TNF-α, IL-6, IL-1β, IL-4 and IL-10 levels in BAL were determined using ELISA. Values are expressed as mean ± SD; ^**^P<0.01 vs. normal control and sham operation groups. ^#^P<0.05, ^##^P<0.01 vs. control group.

CLP caused a significant acute systemic inflammatory response as evidenced by the increased BAL concentrations of the pro-inflammatory mediators TNF-α, IL-1β and IL-6. The presence of XBJ reduced these three pro-inflammatory cytokines. CLP also caused an increase of the BAL concentration of the anti-inflammatory cytokine IL-10. This change in IL-10 and IL-4 concentration was relatively increased by the administration of XBJ (Fig. 7).

### XBJ administration suppresses oxidative stress response

**Figure 8:**
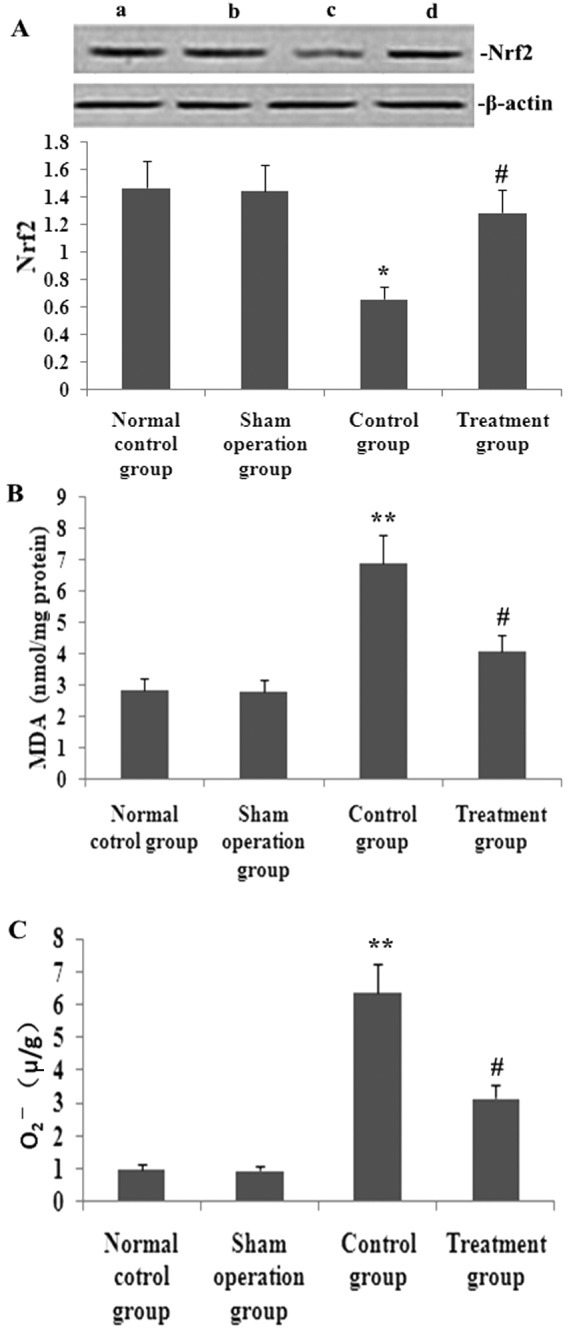
Administration of XBJ attenuated LPS-induced pulmonary oxidative stress response. Groups of mice were challenged with CLP and treated with XBJ 24 h later. The protein expression of (A) Nrf2, (B) MDA and (C) O_2_^−^ weremeasured. All the values are expressed as mean ± SD; ^*^P<0.05, ^**^P<0.01 vs. the normal control and sham operation groups; ^#^P<0.05 vs. control group.

To investigate the effect of XBJ on the oxidative stress response in septic rat lung, the activity of MDA, O_2_^−^ and SOD, and Nrf2 protein expression in lung tissues were measured (Fig. 8). After CLP operation, the expression of Nrf2 protein and the activity of MDA and O_2_^−^ in lung tissues was increased significantly, while the activity of SOD was markedly decreased. However, after the administration of XBJ, the protein expression of Nrf2 and activity of SOD was markedly enhanced, and the activity of MDA and O_2_^−^ in lung tissues was inhibited.

### XBJ administration suppresses VEGF-α expression and secretion

**Figure 9:**
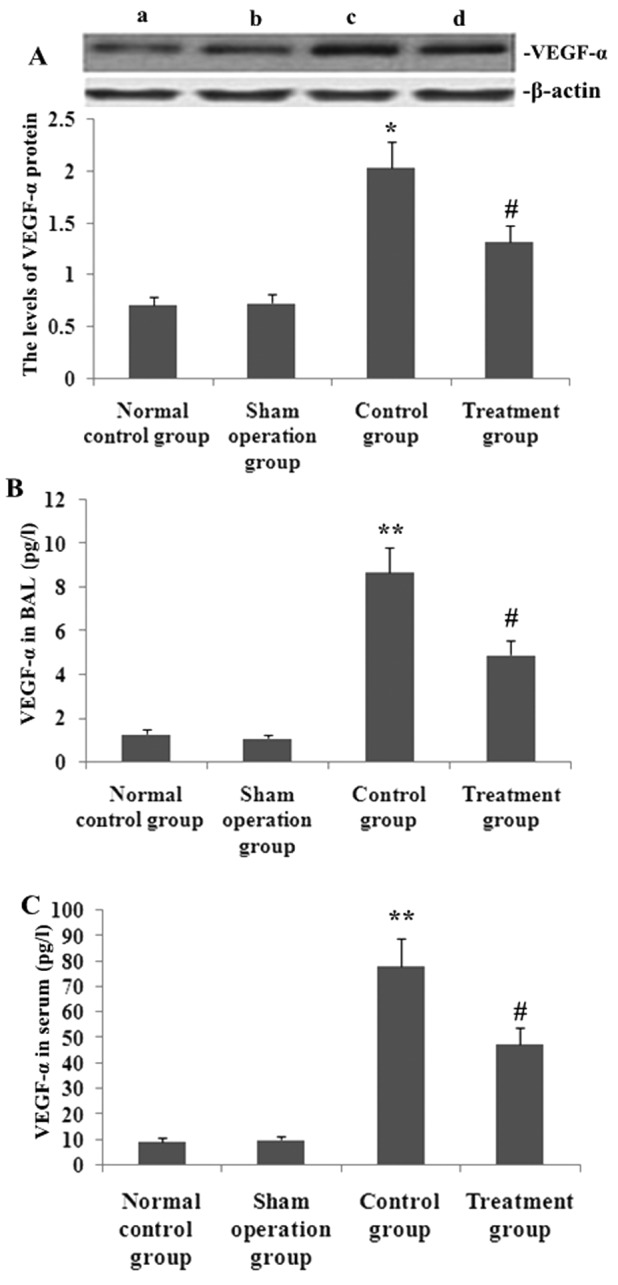
Administration of XBJ reduced the CLP-induced VEGF-α production and expression. Groups of rats were treated as described in Materials and methods. VEGF-α levels in (B) BAL fluid and (A) serum, and (C) the protein expression in lung tissue were determined at 24 h after the CLP challenge. Data are presented as mean ± SD of one experiment consisting of three replicates. ^*^P<0.05, ^**^P<0.01 vs. normal control and sham operation groups; ^#^P<0.05, ^##^P<0.01 vs. control group.

The increase of VEGF-α expression exacerbated pulmonary permeability leakage in septic rats. To analyze the effect of XBJ treatment on VEGF-α expression in lung tissue and secretion in serum and BAL, VEGF-α expression and secretion were measured (Fig. 9). After CLP operation, the expression of VEGF-α in lung tissue and VEGF-α levels in serum and BAL were elevated significantly. However, after XBJ treatment, the expression of VEGF-α in lung tissue and VEGF-α levels in serum and BAL was blocked significantly.

### XBJ administration ameliorates pulmonary permeability

FITC-labeled albumin in BAL, wet/dry lung weight ratio and the water content in lung tissue were reliable parameters of pulmonary permeability. To identify the effect of XBJ administration on pulmonary permeability in septic rats, FITC-labeled albumin in BAL, wet/dry lung weight ratio and the water conten in lung tissue were assayed. As shown in Fig. 11, FITC-labeled albumin in BAL, wet/dry lung weight ratio and the water conteny in lung tissue were increased significantly in CLP-induced rats. However, the increase of FITC-labeled albumin in BAL, wet/dry lung weight ratio and the water content in lung tissue were decreased by XBJ. Therefore, the effects of pulmonary permeability were significantly blocked by XBJ (P<0.05; Fig. 11).

### XBJ administration ameliorates histological acute lung injury

CLP significantly increased lung injury (Fig. 8). The lung tissue was significantly injured with the presence of intra-alveolar exudate, edema, and inflammatory cell infiltrationin the control group compared with that in the treatment group, as evidenced by the increase in the lung injury score (P<0.05; Fig. 7). XBJ significantly attenuated CLP-induced histopathologic changes as evidenced by the decrease in the lung injury score (P<0.05; Figs. 12 and 13).

### XBJ administration ameliorates arterial blood gas in rats with sepsis

The acute lung injury reduced PO2 and PO2/FiO2 in arterial blood in rats with sepsis. To determine the effect of XBJ administration on arterial blood gas in rats with sepsis. PO2 and PO2/FiO2 in arterial blood were measured (Fig. 14). Compared with the CLP group, XBJ administration significantly increased CLP-induced PO2 and PO2/FiO2 (P<0.05). Therefore, the effects of arterial blood gas were significantly improved by XBJ (P<0.05).

## Discussion

The animal model with sepsis using the CLP method is of high stability and repeatability as well as applicability (21), and is currently regarded as the ‘gold standard’ for studies of sepsis. Therefore, this experiment utilized the CLP animal model with sepsis.

Xuebijing (XBJ) is a Chinese medicine compound preparation, consisting of safflower yellow A, tetramethylpyrazine, danshensu and ferulic acid. XBJ has been widely used to treat sepsis and to protect specific organs, regulating the inflammatory response and oxidative stress and improving coagulation and immune function (22,23), all of which are involved in sepsis. XBJ has been injected into patients for 8 years to treat SIRS and MODS induced by infection or ischemia/reperfusion. XBJ has been reported to significantly decrease the serum concentrations of IL-1 and IL-8 in ICU patients (24). Moreover, XBJ injection was found to reduce the secretion of TNF-α and IL-6 and to inhibit SIRS during cardiopulmonary bypass (24). A meta-analysis evaluating the efficacy and safety of XBJ injection in the treatment of sepsis showed that XBJ injection may decrease 28-day mortality rates, complication rates, average length of hospital stay and APACHE II scores (25). The clinical efficacy of XBJ in sepsis suggested that this agent can reduce the secretion of inflammatory cytokines by LPS-activated mononuclear cells/macrophages (26). Therefore, we investigated the effects of XBJ pretreatment on lung injury induced by CLP in rats, and whether the mechanisms underlying these effects were correlated with pro-inflammatory cytokines by induction of CLP.

Sepsis is caused by a systemic response to infection, which is characterized by elevated levels of proinflammatory cytokines such as TNF-α, IL-1β, IFNγ and IL-6 in the circulation or in inflamed tissues (27,28). While the relevance of proinflammatory cytokines to vascular leakage has previously been established (28), the molecular mechanism causing vascular leakage is not fully understood (27). Moreover, no specific therapy is available to treat this pathology. In the present study, XBJ improved lung capillary leakage by inhibiting the generation of inflammatory mediators, such as IL-6 and TNF-α by upregulating Tollip expression. Evidence suggest that neutrophils play a critical role in ALI. When ALI occurs, neutrophils adhere to the injured capillary endothelium and migrate into the air spaces (29). In the present study, mice exposed to LPS exhibited massive recruitment of inflammatory cells, including neutrophils and macrophages to the airways. By contrast, pre-administration with XBJ significantly inhibited the LPS-induced increase in the numbers of total cells, neutrophils and macrophages in the BAL fluid. There was substantial infiltration of neutrophils in mice with LPS-induced ALI, consistent with MPO activity analysis of the lung, which reflects neutrophil and macrophage diapedesis (30) and histological analysis of the lung. XBJ successfully attenuated lung MPO activity and reduced tissue neutrophilia. Additionally, the lung histological examination revealed a decrease in the number of total cells and neutrophils. These findings indicate that the protective effect of XBJ on lung vascular leakage induced by LPS depends on the attenuation of inflammatory cell sequestration and migration into the lung tissue.

A limited number of transcription factors regulate inflammatory pathways, with the most important transcriptional regulator being NF-κB (31). Following activation by a wide array of mediators, including cytokines, bacterial toxins or oxidative stress, the signal transduction cascade is initiated. Activated NF-κB can translocate to the nucleus and bind to the promoters of proinflammatory genes, leading to enhanced gene expression and amplification of the inflammatory response. The NF-κB transcription factor belongs to a family of closely related protein dimers that are considered to be key mediators of inducible transcription in the immune system (31). These mediators are typically activated following the stimulation of cells with pro-inflammatory ligands, including cytokines and bacterial antigens (32). Additionally, the NF-κB transcription factor simultaneously regulates the expression of a great number of genes that have important functions in the regulation of immune and inflammatory responses, including cytokines, chemokines, adhesion molecules and other immunoregulatory proteins to protect cells from the potentially damaging effects of inflammation (32,33). Apart from these, a number of other pathways for NF-κB activation such as p38MAPK and Toll-like receptors signaling pathway have also been elucidated (34). Our results confirm XBJ was able to suppress NF-κB transcriptional activation and led to the concomitant attenuation of inflammatory cytokine expression in the lung following experimental sepsis in the rats, while XBJ increased Tollip expression and impaired NF-κB activation.

Negative regulators were identified at almost every step of the TLR signaling cascade (35–37) and include Toll-interacting protein (TOLLIP, also known as IL-1RAcPIP), A20 (38), single Ig IL-1-receptor (SIGIRR, also termed TIR8) (39), and interleukin-1 receptor-associated kinase 3 (IRAK-3, also known as IRAK-M) (40). TOLLIP is a 274 amino acid protein with highly conserved C2 (amino acids 54–186, similar to that found in PI-specific phospholipase C-d1I) and C-terminal UBA (ubiquitin-associated) domains. Toll-interacting protein (Tollip) is an adaptor protein that acts as an inhibitory factor in TLR signaling cascades (35). When activated by IL-1 or lipopolysaccharide stimulation, Tollip associates with the cytoplasmic TIR domains of IL-1 receptor (IL-1R), as well as TLR2 and TLR4 (36,37). Tollip also interacts with IL-1 receptor-associated kinase 1 (IRAK1) and suppresses its kinase activity (9). Therefore, in the absence of infection, Tollip probably maintains immune cells in a resting state and terminates IL-1R- and TLR-induced inflammatory pathways via the suppression of IRAK1 activity (41). In this regard, Tollip resembles IL-1R-associated kinase M, which also acts as a negative regulator in IL-1β signaling. IL-1R-associated kinase M associates with IRAK1 by blocking IL-1R-associated kinase 4 recruitment, thereby inhibiting IRAK1 phosphorylation and/or activation (42). IRAK1 is an adaptor for the Toll/IL-1R receptor signaling complex. Following IL-1 stimulation, formation of the heterodimeric receptor complex creates a scaffold for the association of MyD88 and Tollip (40). IRAK1 is then recruited to the active receptor complex. Concomitantly, IL-1R-associated kinase 4 is recruited to the receptor complex and may phosphorylate IRAK1, thus, initiating further autophosphorylation of IRAK1. Hyperphosphorylated IRAK1 dissociates from the receptor complex, presumably dimerizes, and binds to TNF receptor-associated factor 6 (TRAF6). IRAK1 binding to TRAF6 functions together with Ubc13/Uev1A to catalyze Lys63-polyubiquitination of IRAK1. Active IRAK1 subsequently causes the dimerization and polyubiquitination of TRAF6, which activates the downstream component, transforming growth factor-β activated kinase 1 (TAK1). TAK1 subsequently phosphorylates several regulatory kinases in different downstream signaling pathways, which ultimately leads to the production and release of multiple cytokines via NF-κB activation (43). However, *in vivo* murine knockout models demonstrated that TOLLIP induced proinflammatory pathways, in contrast to *in vitro* experiments (11). In the experiment, CLP stimulated TLR4, IRAK1, TRAF6 and NF-κB activation, and promoted Tollip expression. However, the administration of XBJ increased Tollip expression, blocked TLR4, IRAK1, TRAF6, while NF-κB activation decreased inflammatory cytokine production. Therefore, XBJ inhibited TLR4, IRAK1, TRAF6 and NF-κB activation by facilitating Tollip overexpression.

Sepsis is associated with oxidative and nitrative stress. It is well known that oxidative stress is a fundamental component of pathogenesis of acute lung injury (44). MDA is the final product of peroxidation (44). SOD and GSH are important protective antioxidants against oxidants and electrophilic compounds, which have been shown to be critical to the antioxidant defences of the lung, particularly in protecting epithelium and endothelium from oxidant injury and inflammation (45,46). CLP results in marked oxidative stress which is demonstrated by an increase of MDA and depletion of SOD and GSH in the lungs. Oxidative stress increases the permeability of the blood gas barrier and results in the formation of lung oedema (46). Nuclear factor-erythroid 2 related factor 2 (Nrf2) is a member of the family of cap‘n’collar basic leucine zipper transcription factors (47) and, although it is ubiquitously expressed throughout the lung, it is found predominantly in the epithelium and alveolar macrophages (AM) (48). Activation of the majority of antioxidant and defense genes are regulated by Nrf2 through binding to antioxidant response elements (AREs) (47). It has been recently reported that the antioxidant pathway controlled by Nrf2 is pivotal for protection against the development of influenza virus-induced pulmonary inflammation and lung injury in mice *in vivo* under oxidative conditions (49). Our results have shown that XBJ treatment enhances Nrf2 activity, blocks and alleviates oxidative stress, and alleviates lung permeability, which is partially contributed to the protection of XBJ against lung injury by CLP through the upregulation of Tollip activity.

VEGF is crucial in the known microvascular permeability inducers, and histamines can have a 50,000-fold effect when the concentration is <1 nmol/l. Such an effect cannot be inhibited by antihistamines and PAF inhibitors as well as other inflammatory inhibitors (48). VEGF activates the Src-Vav2-Rac1-PAK pathway, leading to phosphorylation and disassembly of VE cadherin (50). The latter is an adherens junction protein that also contributes to endothelial barrier integrit. Capillary permeability increase is a key factor in the development of sepsis, while VEGF is the key molecule for controlling vascular permeability, which is a potential factor that leads to inflammation-related capillary permeability (51). In the present study, an increase in the expression of proinflammatory media can promote the expression of VEGF, and elevate lung capillary permeability, however, the expression of VEGF was inhibited by XBJ by an increase in Tollip expression, which ameliorates vascular permeability.

**Figure 10:**
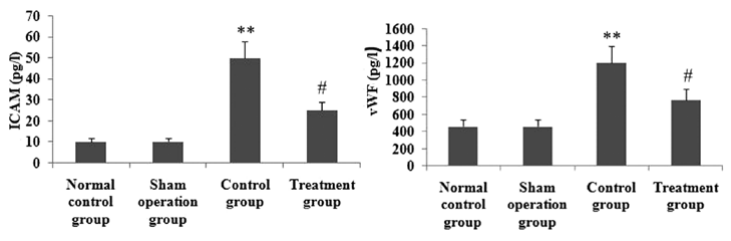
XBJ decreased the CLP-induced ICAM and vWF production. Groups of rats were treated as described in Materials and methods and then ICAM and vWF levels in serum were determined at 24 h after the CLP challenge. Data are presented as mean ± SD of one experiment consisting of three replicates. ^*^P<0.05, ^**^P<0.01 vs. the normal control and sham operation groups; ^#^P<0.05, ^##^P<0.01 vs. control group.

**Figure 11:**
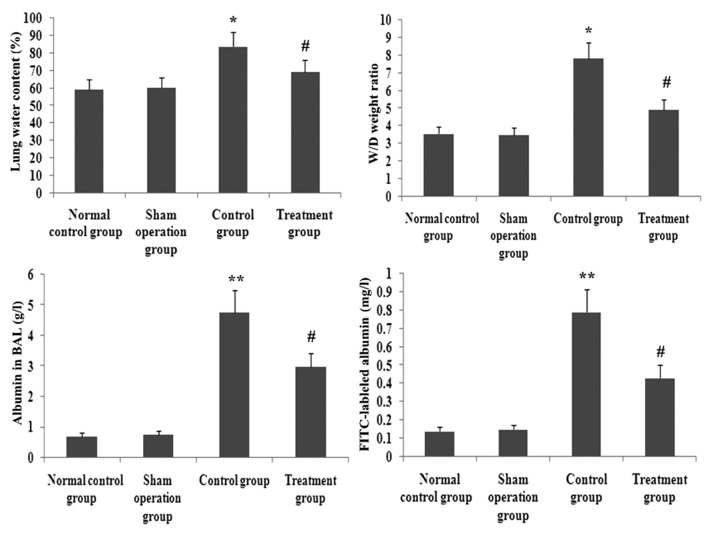
XBJ reduced the CLP-induced lung permeability. Groups of rats were treated as described in Materials and methods. FITC-labeled albumin in the BAL fluid, wet/dry lung weight ratio and lung water content in lung tissue were determined at 24 h after the CLP challenge. Data are presented as mean ± standard deviation of one experiment consisting of three replicates. ^*^P<0.05, ^**^P<0.01 vs. normal control and sham operation groups; ^#^P<0.05, ^##^P<0.01 vs. control group.

**Figure 12:**
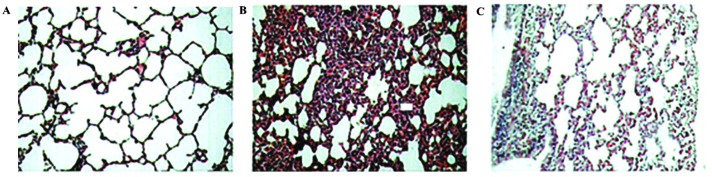
Administration of XBJ ameliorated the histopathologic changes of lung in CLP-ALI mice. Groups of mice were treated as described in Materials and methods. Histological evaluation of the therapeutic potential of XBJ on LPS-induced lung injury in mice was analyzed at 24 h after the LPS challenge. Representative images of hematoxylin and eosin-stained lung sections from four experimental groups. (A) Sham operation, (B) control and (C) treatment groups.

**Figure 13:**
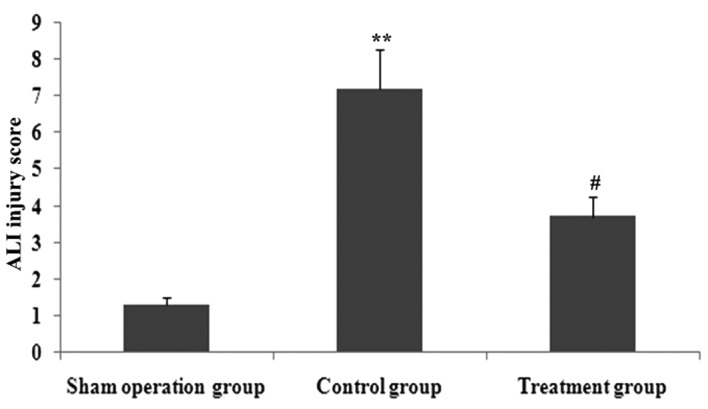
Administration of XBJ decreased the acute lung injury histopathologic score of lung in CLP-ALI mice. Groups of mice were treated as described in Materials and methods and a histological evaluation of the therapeutic potential of salidroside on LPS-induced lung injury in mice was analyzed at 24 h after LPS challenge. Lung injury score was determined. Data are presented as mean ± SD of one experiment consisting of three replicates. The ALI pathology score is expressed as mean ± SD; ^**^P<0.01 vs. the normal control and sham operation groups; ^#^P<0.05 vs. control group.

**Figure 14:**
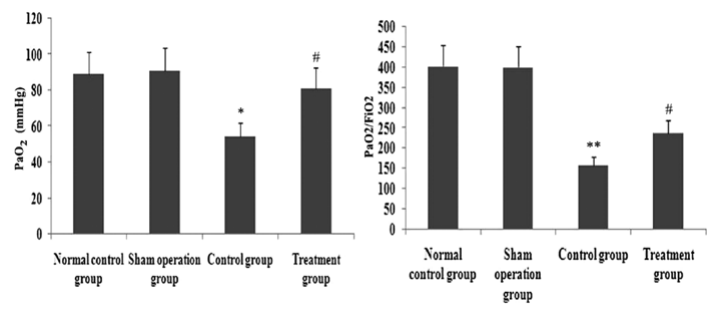
Effect of administration of XBJ on LPS-induced arterial blood gas in the four rat groups. Groups of mice were challenged with CLP and treated with XBJ 24 h later the arterial blood gas were measured. Values are expressed as mean ± SD; ^*^P<0.05, ^**^P<0.01 vs. the normal control and sham operation groups; ^#^P<0.05 vs. control group.

vWF is a substantially large plasma glycoprotein synthesized in endothelial cells and megakaryocytes, the primary function of which is to attach platelets to sites of blood vessel injury (52). vWF is often described as ‘a marker of endothelial activation’ (53). Although in many cases, this may be true, is it only part of its function, especially given the effect of inflammation and oxidation in preventing cleavage and enhancing the function of important adhesive molecules, such as ICAM. In the setting of sepsis, endothelial cells showed a marked increase in surface ICAM-1 and expression of VCAM-1 on endothelial cells. Progressive increases in levels of vascular ICAM-1 occurred following sepsis, and such changes were identified in numerous organs (54). In this experiment, we also found that CLP increased the secretion of vWF and ICAM-1 in serum, and microvascular permeability in lung tissue deteriorated. However, XBJ reduced the secretion of vWF and ICAM-1 in serum, while the increase of microvascular permeability in lung tissue was inhibited by XBJ (Fig. 10).

Vascular leakage is a critical pathological process in sepsis-induced ALI (27). It permits plasma protein and leukocyte extravasation, leading to edema and inflammatory reactions in the inflamed tissues (27). Edema causes tissue hypoxia. Leukocytes, such as neutrophils, cause tissue damage through the excessive production of free radicals and proteases. Vascular leakage is thus a promising target for therapeutic treatment. Vascular leakage in various organs is a characteristic pathological change in sepsis (55). In the present study, we first evaluated the W/D ratio of the lung. The results show that salidroside treatment attenuates the development of pulmonary edema, as determined by the significant decrease in lung W/D ratio. As another index of ALI by LPS, we measured total protein concentration in the BAL fluid, which indicates epithelial permeability and pulmonary edema. FITC-labeled albumin, a macromolecular marker, is widely used to evaluate pulmonary microvascular permeability (50). We also measured FITC-labeled albumin. As expected, LPS instillation was found to cause a significant increase in BAL fluid protein concentration, lung W/D ratio and FITC-labeled albumin. LPS-induced increases in total protein in the BAL fluid, lung W/D ratio and FITC-labeled albumin were inhibited by XBJ.

An arterial blood gas (ABG) is a blood test performed using blood from an artery. An ABG is a test that measures the arterial oxygen tension (PaO2), carbon dioxide tension (PaCO2) and acidity (pH). In addition, arterial oxyhemoglobin saturation (SaO2) can be determined. Such information is vital when caring for patients with critical illness or respiratory disease. As a result, the ABG is one of the most common tests performed on patients in intensive care units (ICUs). The arterial oxygen tension and oxygenation index(PaO2/FiO2) are two important indicators to judge acute lung injury. In this experiment, the results showed that the reduction of the arterial oxygen tension and oxygenation index was enhanced by XBJ. We also found that XBJ significantly attenuated CLP-induced pathologic changes as evidenced by a decrease in the lung injury score.

In conclusion, although XBJ has been reported for its decrease of the lung capillary leakage properties in clinical practice, the inner molecular biological mechanisms have not been sufficiently investigated. The study indicates XBJ may have an impact on decreasing lung capillary leakage by upregulating Tollip expression and inhibiting lung inflammatory reponse and oxidant stress, and alleviating lung permeability, in order to induce CLP-induced lung injury.

## Figures and Tables

**Table I:** Primer sequences for the genes to validate the microarray analysis by RT-PCR.

Gene	Primer	Product (bp)
Tollip	mRNA F: 5′-GGACAACGGTCAGCGACGCA-3′ R: 5′-CATAGCCCAGACGCAGGCGG-3′	272
NF-κB mRNA	F: 5′-GCACGGATGACAGAGGCGTGTATAAGG-3′ R: 5′-GGCGGATGATCTCCTTCTCTCTGTCTG-3′	420
IRAK1 mRNA	F: 5′-ATGCCTATGTTCATCGTGAAC-3′ R: 5′-GGCTACGACGAAGGTGGAAC-3′	341
TLR4 mRNA	F: 5′-CCAGGAAGGCTTCCACAAGA-3′ R: 5′-AATTCGACCTGCTGCCTCAG-3′	351
TRAF6 mRNA	F: 5′-AAGATTGGCAACTTTGGGATG-3′ R: 5′-GTGGGATTGTGGGTCGCTG-3′	331
β-actin	F: 5′-TGGAATCCTGTGGCAGTCCAGT-3′ R: 5′-TAAAACGCAGCTCAGTAACAG-3′	349

## Abbreviations

XBJ: Xuebijing injection
Tollip: Toll-interacting protein
IRAK1: interleukin-1 receptor-associated kinase 1
TRAF6: TNF receptor-associated factor 6
NF-κB: nuclear factor κB
IL-6: interleukin-6
TNF-α: tumour necrosis factor-α
CLP: cecal ligation and puncture
RT-PCR: reverse transcription polymerase chain reaction
BAL: bronchoalveolar lavage fluid
TLR4: Toll-like receptor 4
HO-1: heme oxygenase-1
MPO: myeloperoxidase
MDA: malondialdehyde
SOD: superoxide dismutase
